# The contributions of positive outgroup and negative ingroup evaluation to implicit bias favoring outgroups

**DOI:** 10.1073/pnas.2116924119

**Published:** 2022-09-26

**Authors:** Jimmy Calanchini, Kathleen Schmidt, Jeffrey W. Sherman, Samuel A. W. Klein

**Affiliations:** ^a^Department of Psychology, University of California, Riverside, CA 92521;; ^b^Department of Psychology, Southern Illinois University, Carbondale, IL 62901;; ^c^Department of Psychology, University of California, Davis, CA 95616

**Keywords:** intergroup bias, outgroup bias, implicit attitudes, racism, homophobia

## Abstract

People sometimes prefer groups to which they do not belong (outgroups) over their own groups (ingroups). Many long-standing theoretical perspectives assume that this outgroup favorability bias primarily reflects negative ingroup evaluations rather than positive outgroup evaluations. To examine the contributions of negative ingroup versus positive outgroup evaluations to outgroup bias, we examined participants’ data (total *n* > 879,000) from Implicit Association Tests [A. G. Greenwald, D. E. McGhee, J. L. K. Schwartz, *J. Pers. Soc. Psychol.* 74, 1464–1480 (1998)] measuring intergroup attitudes across four social domains in exploratory and preregistered confirmatory analyses. Process modeling [F. R. Conrey, J. W. Sherman, B. Gawronski, K. Hugenberg, C. J. Groom, *J. Pers. Soc. Psychol.* 89, 469–487 (2005)] was applied to the responses of participants who demonstrated implicit outgroup bias to separately estimate the contributions of negative ingroup and positive outgroup evaluations. The outgroup biases of lower-status group members (i.e., Asian, Black, gay and lesbian, and older people) consistently reflected greater contributions of positive outgroup evaluations than negative ingroup evaluations. In contrast, the outgroup biases of higher-status group members (i.e., White, straight, and younger people) reflected a more varied pattern of evaluations. We replicated this pattern of results using explicitly measured intergroup evaluations. Taking these data together, the present research demonstrates a positive–negative asymmetry effect of outgroup bias, primarily among members of lower-status groups.

## Significance

Many long-standing theoretical traditions assume that preferences for outgroup members over ingroup members reflect internalized negativity toward the ingroup, especially among members of lower-status groups. Process modeling of implicit outgroup biases suggested otherwise. Among lower-status group members examined here, positivity toward the outgroup contributed more to outgroup biases than negativity toward the ingroup. The pattern of results was mixed among high-status group members with implicit outgroup biases. These findings suggest that members of lower-status groups who prefer higher-status groups over their own groups do not necessarily harbor group self-hatred; instead, they primarily evaluate higher-status outgroups positively.

Intergroup bias can be based on feelings toward the ingroup, feelings toward the outgroup, or both. A wide range of theoretical perspectives converge on the conclusion that ingroup favorability bias primarily reflects positive evaluations of the ingroup rather than negative evaluations of the outgroup (e.g., refs. 1–5). This positive–negative asymmetry effect (5) is supported by empirical evidence that ingroup bias typically reflects love of “us” more than it reflects hatred of “them” (e.g., refs. 6 and 7). In contrast, outgroup favorability bias is often assumed to primarily reflect negative evaluations of the ingroup. Early intergroup relations theories posited that members of disadvantaged groups who face persistent social hostility ultimately internalize their putative inferiority and manifest “group self-hatred” (1, 8–10). More recent perspectives have identified conditions and motivations under which lower-status group members may favor higher-status outgroups [e.g., to promote hierarchies (11) or to see the status quo as fair (12)], but they remain relatively agnostic on the primacy of negative ingroup versus positive outgroup evaluations. Outgroup favorability bias is normatively rarer among members of higher-status groups; accordingly, theoretical perspectives on intergroup bias are also largely silent on the mechanisms underlying the biases of higher-status group members who favor lower-status outgroups. However, anecdotally, members of higher-status groups who are perceived to favor lower-status outgroups are often accused of internalizing negative evaluations of their ingroup (e.g., “White guilt”), as well as positive evaluations of the outgroup (e.g., White people who appropriate Black culture). The present research aims to address this gap in our understanding of intergroup bias by examining the extent to which outgroup favorability bias is characterized by positive versus negative evaluations.

## Implicit vs. Explicit Outgroup Bias.

Extensive research has established robust ingroup favorability biases across a variety of ingroup–outgroup category distinctions, including those based on age, race, ethnicity, nationality, sexuality, and religious affiliation (13, 14). Outgroup favorability bias, on the other hand, has been examined much less in the intergroup evaluation literature, perhaps because it is relatively more challenging to measure. Members of low-status groups often feel intense pressure to show ingroup pride, so they might be reluctant to openly display outgroup bias (12). Indeed, when intergroup bias is measured using explicit (i.e., direct) measures, members of low-status groups generally demonstrate ingroup bias (12, 15). However, intergroup bias can also be measured using implicit (i.e., indirect) measures that infer evaluations from the speed and accuracy of responses, rather than the contents of responses, per se.* Relative to explicit measures, implicit measures reduce the influence of social desirability and self-presentation concerns. By obscuring what is being measured or making responses difficult to control, implicit measures are thus less subject to intentional manipulation (16). Under such constrained response conditions, members of low-status groups often demonstrate implicit outgroup bias, in preference of higher-status groups (12, 15, 17). That said, members of higher-status groups normatively demonstrate ingroup bias on both explicit and implicit measures (12, 15).

Given the focus in the present research on outgroup bias and the elusive nature of outgroup bias on explicit measures, we focus here primarily on implicitly measured outgroup bias. However, this measurement approach also comes with limitations. Implicit intergroup bias is traditionally operationalized in terms of relative preference for one group versus the other (18). Such relative operationalizations obscure the independent contributions of positive versus negative evaluations of either group to implicit intergroup bias. Consequently, we employed formal modeling techniques to disentangle the contributions of positive and negative evaluations to outgroup bias.

## Research Impetus.

The aim of the present research was to examine the relative contributions of positive and negative evaluations to implicit outgroup bias. To do so, we applied the Quadruple Process model (Quad model) (19) to responses on Implicit Association Tests (IAT) (20) from two participant populations (undergraduates, visitors to an internet demonstration website) and three content domains (race, sexual orientation, age). We first investigated this research question in an exploratory sample before seeking to replicate the pattern of results with a preregistered confirmatory sample. The patterns of results persisted across a variety of analytic approaches and operationalizations of outgroup bias (preregistration available at https://osf.io/pf8cd/).^†^

## Methods

Below, we report all sample size determinations, data exclusions, manipulations, and measures (21). All data and materials are available online at the Open Science Framework (*Data, Materials, and Software Availability*).

### Participants.

We report descriptive statistics for all participant samples in Table 1. In both the exploratory and confirmatory analyses, we primarily relied on very large samples of internet participants. Not only do these samples provide very good statistical power, but they are also more diverse than traditional samples of university undergraduates on the dimensions of race/ethnicity, age, political orientation, socioeconomic status, and educational background. Additionally, to further increase the generalizability of our findings, both sets of analyses also include one university sample each. To the extent that we observe consistent results across different participant populations, we can have greater confidence in the generalizability of these findings. The Institutional Review Board of the University of California approved the studies that relied on university participants, and the Institutional Review Board of the University of Virginia approved the studies that relied on internet participants. Participants provided informed consent electronically before participating in the study.

**Table 1.** Descriptive statistics by participant sample as a function of group status and direction of implicit bias

Sample	Total *n*	Biased toward outgroup		Biased toward ingroup		Comparison Cohen’s *d*
		*n* (% of total)	*D*-score mean (SD)	*n* (% of total)	*D*-score mean (SD)	
Lower-status ingroups						
Exploratory sample						
Asian people	96	35 (36.5)	0.48 (0.24)*	34 (35.4)	−0.46 (0.22)*	2.90*
Black people	35,045	12,338 (35.2)	0.45 (0.23)*	14,034 (40.0)	−0.47 (0.23)*	2.98*
Gay/lesbian people	63,535	17,015 (26.8)	0.43 (0.22)*	31,685 (49.9)	−0.50 (0.25)*	3.11*
Older people	1,683	1,309 (77.8)	0.63 (0.30)*	136 (8.1)	−0.32 (0.15)*	3.89*
Confirmatory sample						
Asian people	207	74 (35.7)	−0.04 (0.35)	100 (48.3)	−0.20 (0.34)*	3.96*
Black people	5,000	2,434 (48.7)	0.36 (0.40)*	1,581 (31.6)	−0.03 (0.43)*	2.50*
Gay men (g/s)	5,000	1,697 (33.9)	−0.04 (0.41)*	2,362 (47.2)	−0.31 (0.38)*	3.91*
Gay men (l/s)	5,000	1,720 (34.4)	−0.01 (0.41)	2,315 (46.3)	−0.29 (0.39)*	3.80*
Gay men (b/s)	5,000	1,690 (33.8)	−0.03 (0.40)*	2,367 (47.3)	−0.31 (0.38)*	3.91*
Lesbian women (g/s)	5,000	1,696 (33.9)	−0.02 (0.41)	2,404 (48.1)	−0.33 (0.39)*	3.88*
Lesbian women (l/s)	5,000	1,319 (26.4)	−0.16 (0.41)*	2,814 (56.3)	−0.46 (0.36)*	4.70*
Lesbian women (b/s)	5,000	1,523 (30.5)	−0.08 (0.41)*	2,612 (52.2)	−0.38 (0.37)*	4.25*
Older people	5,000	2,788 (55.8)	0.55 (0.37)*	1,073 (21.5)	0.26 (0.40)*	1.90*
Higher-status ingroups						
Exploratory sample						
White (vs. Asian) people	38	2 (5.3)	−0.29 (0.07)	30 (78.9)	0.70 (0.24)*	1.46*
White (vs. Black) people	271,569	25,461 (9.4)	−0.37 (0.21)*	202,638 (74.6)	0.58 (0.26)*	1.79*
Straight people	472,712	57,648 (12.2)	−0.41 (0.24)*	338,696 (71.6)	0.60 (0.28)*	1.70*
Younger people	88,574	5,054 (5.7)	−0.34 (0.17)*	72,036 (81.3)	0.62 (0.27)*	1.67*
Confirmatory sample						
White (vs. Asian) people	101	33 (32.7)	0.17 (0.48)	57 (56.4)	0.40 (0.31)*	2.38*
White (vs. Black) people	5,000	1,245 (24.9)	0.13 (0.41)*	2,779 (55.6)	0.42 (0.38)*	2.23*
Straight men (g/s)	5,000	1,156 (23.1)	0.23 (0.42)*	2,986 (59.7)	0.44 (0.38)*	2.09*
Straight men (l/s)	5,000	1,105 (22.1)	0.21 (0.43)*	3,067 (61.3)	0.46 (0.39)*	2.04*
Straight men (b/s)	5,000	1,105 (22.1)	0.23 (0.43)*	3,012 (60.2)	0.45 (0.39)*	2.05*
Straight women (g/s)	5,000	1,266 (25.3)	0.07 (0.42)*	2,836 (56.7)	0.36 (0.38)*	2.37*
Straight women (l/s)	5,000	1,566 (31.3)	−0.03 (0.43)	2,504 (50.1)	0.29 (0.41)*	2.60*
Straight women (b/s)	5,000	1,358 (27.2)	0.05 (0.42)*	2,721 (54.4)	0.33 (0.39)*	2.45*
Younger people	5,000	1,143 (22.9)	0.28 (0.37)*	2,968 (59.4)	0.52 (0.34)*	2.02*

For effect-size calculations, sample means were compared to zero in one-sample *t* tests, and ingroup-favoring and outgroup-favoring means were compared to one another in paired samples *t* tests. “b/s” refers to an IAT with gay and lesbian stimuli along with straight stimuli; “g/s” refers to an IAT with gay/straight stimuli; “l/s” refers to an IAT with lesbian/straight stimuli.

^*^Effects are reliably different from zero at *P* < 0.01.

### Race.

#### Asian vs. White.

Participants were American undergraduates who completed an IAT in which they categorized pleasant and unpleasant words along with pictures of Asian and White people. We define East Asian participants as members of the normatively lower-status group, and White participants as members of the normatively higher-status group.

#### Black vs. White.

Participants were visitors to the North American Project Implicit demonstration website who completed an IAT in which they categorized pleasant and unpleasant words along with pictures of Black and White people. We define Black participants as members of the normatively lower-status group, and White participants as members of the normatively higher-status group.

### Sexual Orientation.

Participants were visitors to the North American Project Implicit demonstration website who completed an IAT in which they categorized pleasant and unpleasant words along with pictures representing gay/lesbian and straight relationships. We define gay and lesbian participants as members of the normatively lower-status group, and straight participants as members of the normatively higher-status group.

### Age.

Participants were visitors to the North American Project Implicit demonstration website who completed an IAT in which they categorized pleasant and unpleasant words along with pictures of old and young people. We operationalized each age group based on the thresholds used by Gonsalkorale et al. (22) and considered participants at least 65 y of age to be older and participants between the ages of 21 and 40 y to be younger. In the exploratory sample, older participants’ mean_age_ = 69.73 y, SD_age_ = 4.62 y, and younger participants’ mean_age_ = 27.72 y, SD_age_ = 5.68 y. In the confirmatory sample, older participants’ mean_age_ = 69.33 y, SD_age_ = 4.41, and younger participants’ mean_age_ = 27.67 y, SD_age_ = 5.85 y. We define older participants as members of the normatively lower-status group, and younger participants as the normatively higher-status group.

### Materials and Measures.

All participants completed an IAT that began with two 20-trial practice blocks, in which they discriminated pleasant from unpleasant words, and the ingroup from the outgroup, respectively. The third and fourth blocks were critical blocks consisting of 20 and 40 trials, respectively. In these blocks, participants pressed one key whenever they saw the ingroup or a pleasant word and another key whenever they saw the outgroup or an unpleasant word. In the fifth 20-trial block, the keys used to categorize the ingroup and outgroup were switched, and participants practiced discriminating the ingroup and outgroup using the new key assignments. The sixth and seventh blocks were critical blocks consisting of 20 and 40 trials, respectively. These blocks were identical to the third and fourth blocks, except participants pressed one key whenever they saw the outgroup or a pleasant word and another key whenever they say the ingroup or an unpleasant word. Group and attribute labels remained on the top left and top right of the screen throughout the task, while stimulus pictures and words appeared at the center of the screen. A red “X” appeared whenever participants made an error, and they were required to correct the error before moving onto the next trial. Latencies were recorded to the correct response, and accuracies recorded the first response. Participants were instructed to make their classifications as quickly and accurately as possible. With the exception of the Asian/White IAT in the exploratory sample, the order of the critical blocks was randomized between participants. The critical blocks of the sexuality IAT for our confirmatory sample included only 20 trials each.

Project Implicit hosts three different versions of the sexuality IAT: one consists of stimuli representing gay (male) and straight relationships; another consists of stimuli representing lesbian (female) and straight relationships; and a third consists of stimuli representing gay, lesbian, and straight relationships. In the exploratory analyses we did not distinguish among participants' responses to the three types of sexuality IATs, and a puzzling pattern of results emerged. Consequently, in the confirmatory analyses we distinguished participants’ responses among the three types of IATs in an attempt to clarify these findings.

## Results

### Modeling.

The Quad model is a multinomial processing tree model (23) that estimates the contributions of latent cognitive processes based on the frequency of correct and incorrect responses. According to the Quad model, four qualitatively distinct processes influence implicit task performance: activation of associations (AC), detection of correct responses (D), overcoming bias (OB), and guessing (G). The AC parameter is most relevant to the present investigation of outgroup bias, as it refers to the degree to which evaluative associations are activated when responding to a stimulus. All else equal, stronger associations are more likely to be activated and to influence responses.

**Fig. 1.**
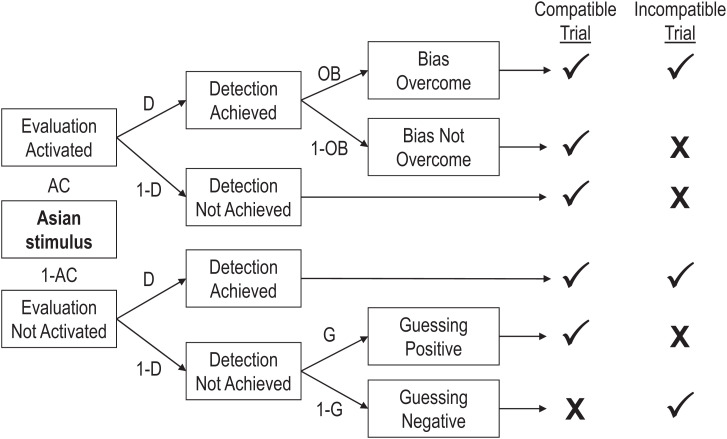
A portion of the Quad model. Each path represents a likelihood. Parameters with lines leading to them are conditional upon all preceding parameters. The table on the right side of the figure depicts correct (✔) and incorrect (✘) responses as a function of process pattern.

The structure of the Quad model is depicted as a processing tree in Fig. 1. In the tree, each path represents a likelihood. Parameters with lines leading to them are conditional on all preceding parameters. For example, OB is conditional on both AC and D. The relationships described by the model form a system of equations that predicts the numbers of correct and incorrect responses in different conditions (e.g., compatible and incompatible blocks). For example, there are three ways in which an incorrect response can be returned on a trial of an Asian/White IAT in which Asian and “pleasant” share a response key for a person with pro-White bias. The first is the likelihood that associations between “Asian” and “unpleasant” are activated (AC), detection of the correct response succeeds (D), and OB fails to overcome the biased association in favor of the correctly detected response (1 − OB), which can be represented by the equation AC × D × (1 − OB). The second is the likelihood that associations are activated (AC) and D fails (1 − D), which can be represented by the equation AC × (1 − D). The third is the likelihood that associations are not activated (1 − AC), D fails (1 − D), and a bias toward responding “unpleasant” (1 − G) produces an incorrect response, which can be represented by the equation (1 − AC) × (1 − D) × (1 − G). As such, the overall likelihood of producing an incorrect response on this trial is the sum of these three conditional probabilities: [AC × D × (1 − OB)] + [AC × (1 − D)] + [(1 − AC) × (1 − D) × (1 − G)]. The respective equations for each response (e.g., correct, incorrect) per item category (e.g., White faces, Asian faces, pleasant words, and unpleasant words in both block types) are then used to predict the observed proportions of errors in each dataset, and the parameter values that best fit the observed responses are interpreted to reflect the likelihood that each process influenced responses.

We estimated two AC parameters for each participant.^‡^ The Quad model assumes a compatibility structure to participants’ responses: namely, that the direction of bias reflects the dominant valences of the underlying associations. Because we focus here on participants who demonstrate outgroup bias, we specified one AC parameter to reflect negative evaluations of the ingroup and another AC parameter to reflect positive evaluations of the outgroup. In the exploratory samples, we aggregated all participants’ responses in each sample and estimated group-level parameters using the maximum-likelihood algorithm implemented in multiTree (24). One advantage of estimating parameters at the group level is that it affords a high degree of statistical power; however, it does not account for heterogeneity among individuals. Consequently, in the confirmatory sample, we estimated parameters using a Bayesian hierarchical approach implemented in the R package TreeBUGS (25). This hierarchical estimation method produces both individual- and group-level parameters.

#### Model fit.

We quantified the extent to which the Quad model adequately fits each dataset in terms of χ^2^ for the exploratory sample, and in terms of *T_1_* (26) for the confirmatory sample. These model fit statistics depend on the total number of observations (i.e., number of participants × number of IAT trials per participant). A large number of total observations provides high statistical power, such that minute deviations from the model can jeopardize model fit when power is high (27). The sizes of the samples reported in this paper vary greatly and are relatively large, on average. We report in *SI Appendix* model fit values for the exploratory and confirmatory samples along with the *w* statistic, which controls for sample size, and thus represents the effect size for the lack of model fit between the actual data and the model’s predicted data (28). Accounting for sample size, model fit was relatively consistent across datasets, and adequate in all cases (28).

#### Operationalizing outgroup bias.

In the exploratory sample, we operationalized participants as demonstrating outgroup bias according to their *D*-scores (18), which are primarily based on response latency. Positive *D*-scores are interpreted to reflect an evaluative preference for the relatively higher-status group, and negative *D-*scores are interpreted to reflect an evaluative preference for the relatively lower-status group. Consequently, in the present research we defined outgroup bias as lower-status participants with *D* > 0.15 and as higher-status participants with *D* < −0.15.^§^

Recognizing the relatively arbitrary nature of the *D*-score cutoff by which we defined outgroup bias in the exploratory sample, we relied on a different operationalization of implicit outgroup bias in the confirmatory sample. Specifically, we applied two different specifications of the Quad model to each participant’s data: one version specified to reflect outgroup bias (i.e., configured to estimate a parameter reflecting negative ingroup evaluations, and a parameter reflecting positive outgroup evaluations), and another version specified to reflect ingroup bias (i.e., configured to estimate a parameter reflecting positive ingroup evaluations, and a parameter reflecting negative outgroup evaluations). We then computed fit index *T_1_* for each model specification for each participant. We defined participants as demonstrating outgroup bias if *T_1_* indicated better fit for the outgroup-bias model specification than for the ingroup-bias model specification. We excluded participants from further analyses for whom neither model fit, or for whom both models fit equally well.

We summarize in Table 1 the proportion of participants from each sample categorized as demonstrating outgroup bias, as well as the average *D*-scores for each sample. The *D-*scores of participants in the confirmatory sample were not used to define their direction of bias, but are presented as a validity check.

#### Planned contrasts.

In order to determine the extent to which positive outgroup versus negative ingroup evaluations contributed to outgroup bias, we conducted a series of planned contrasts to quantify the difference between the outgroup-pleasant AC parameter and the ingroup-unpleasant AC parameter.

In the exploratory sample, conducting planned contrasts was a multistep process in which we first fit a baseline model in which both AC parameters were estimated freely. Subsequently, for each group of participants we fit a model in which the outgroup-pleasant AC parameter was constrained to be equal to the ingroup-unpleasant AC parameter. Without a significant decline in model fit under this parameter constraint, we cannot infer the two parameters are different from one another. However, if fit significantly declines under this parameter constraint, we can infer that the two parameters are different from one another. The magnitude of the difference between AC parameters is quantified in terms of change in model fit (i.e., Δχ^2^). We applied two additional constraints in order to determine whether each parameter can be reliably distinguished from zero. To do so, we separately constrained each AC parameter to be equal to zero. Without a significant decline in model fit, that constrained AC parameter cannot be inferred to be different from zero.

In the confirmatory sample, planned contrasts are relatively more straightforward. For each group of participants, we subtracted the distributions for all posterior samples of the ingroup-unpleasant AC parameter from the distributions for all posterior samples of the outgroup-pleasant AC parameter. In the resulting distribution of mean differences, we infer that the two AC parameters are reliably different from one another if the 95% Bayesian confidence interval (BCI) of the difference does not contain zero, and positive (negative) mean differences indicate that outgroup-pleasant (ingroup-unpleasant) AC estimates are larger than ingroup-unpleasant (outgroup-pleasant) AC estimates. Whereas the maximum-likelihood approach applied to the exploratory sample required model constraint tests to determine whether parameter estimates differed from zero, the hierarchical Bayesian approach applied to the confirmatory sample did not. Instead, parameter estimates in the confirmatory sample can be distinguished from zero if the parameter’s 95% BCI does not contain zero.

**Fig. 2.**
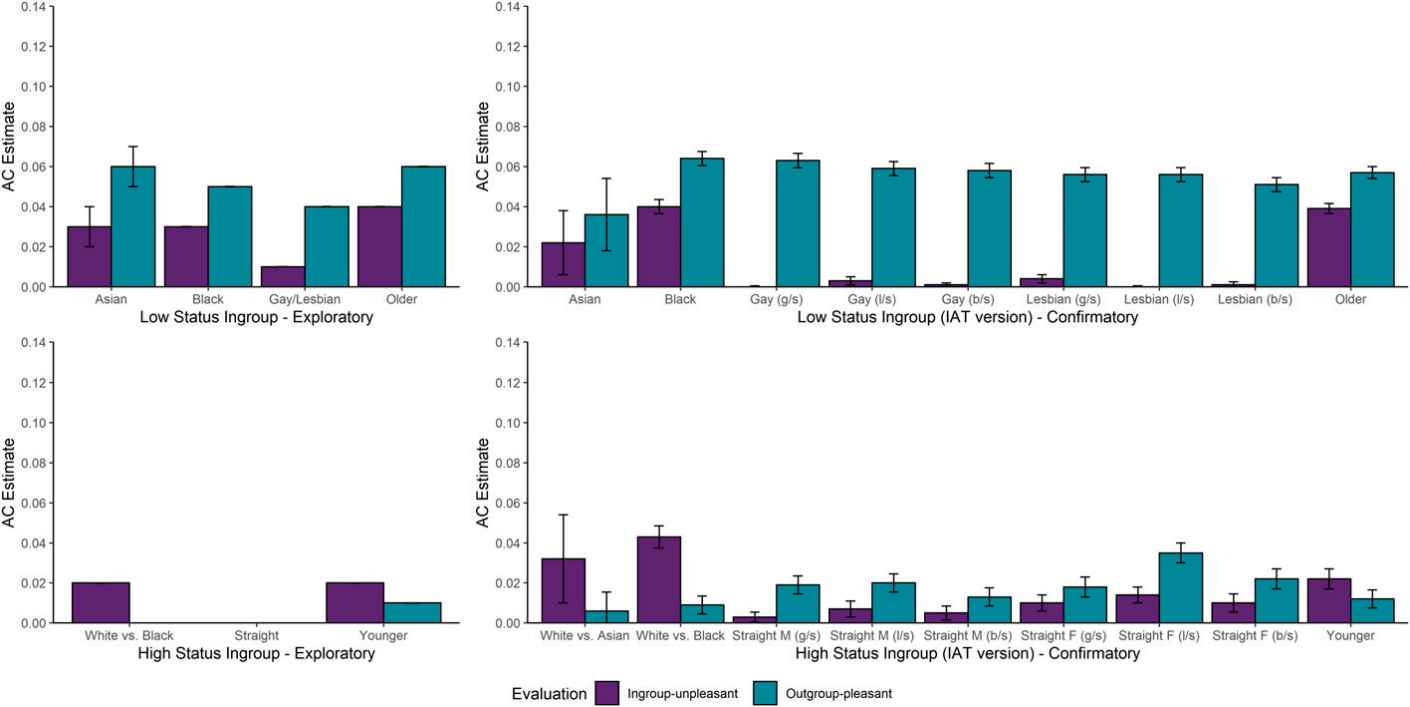
Ingroup-unpleasant and outgroup-pleasant parameter estimates from lower-status participants in the exploratory sample (*Upper Left*), lower-status participants in the confirmatory sample (*Upper Right*), higher-status participants in the exploratory sample (*Lower Left*), and higher-status participants in the confirmatory sample (*Lower Right*). Error bars in the exploratory sample reflect SEs. Error bars in the confirmatory sample reflect 95% BCI.

Because group status plays a prominent role in extant theory about outgroup bias (1, 8–12), we organized our findings according to status (low, high). We depict parameter estimates in Fig. 2, and report planned contrasts for the exploratory and confirmatory samples in Tables 2 and 3, respectively.

**Table 2.** Summary of AC parameter comparisons for outgroup-favoring participants in exploratory sample

Sample	Outgroup+ vs. Ingroup−			Outgroup+ vs. 0			Ingroup− vs. 0		
	Δχ^2^	*P*	*w*	Δχ^2^	*P*	*w*	Δχ^2^	*P*	*w*
Lower-status ingroups									
Asian people	4.18	0.041	0.03	31.24	<0.001	0.08	8.03	0.005	0.04
Black people	256.74	<0.001	0.01	4,825.06	<0.001	0.06	2,041.62	<0.001	0.04
Gay/lesbian people	3,334.08	<0.001	0.04	7,142.57	<0.001	0.06	99.61	<0.001	0.01
Older people	56.96	<0.001	0.02	1,484.19	<0.001	0.10	838.43	<0.001	0.07
Higher-status ingroups									
White (vs. Black) people	944.63	<0.001	0.02	0.00	0.999	0.00	1,197.46	<0.001	0.02
Straight people	0.00	0.999	0.00	0.00	0.999	0.00	0.00	0.999	0.00
Younger people	66.06	<0.001	0.01	23.97	<0.001	0.01	265.41	<0.001	0.02

Outgroup+ refers to positive outgroup evaluations. Ingroup− refers to negative ingroup evaluations.

**Table 3.** Summary of AC parameter comparisons for outgroup-favoring participants in the confirmatory sample

Sample	Outgroup+ vs. Ingroup−	95% BCI
Lower-status ingroups		
Asian people	0.013	[−0.010, 0.036]
Black people	0.024	[0.020, 0.029]
Gay men (g/s)	0.062	[0.059, 0.066]
Gay men (l/s)	0.056	[0.052, 0.060]
Gay men (b/s)	0.057	[0.053, 0.060]
Lesbian women (g/s)	0.052	[0.042, 0.056]
Lesbian women (l/s)	0.055	[0.052, 0.059]
Lesbian women (b/s)	0.049	[0.045, 0.053]
Older people	0.018	[0.014, 0.021]
Higher-status ingroups		
White (vs. Asian) people	−0.026	[−0.050, −0.002]
White (vs. Black) people	−0.035	[−0.041, −0.028]
Straight men (g/s)	0.016	[0.010, 0.021]
Straight men (l/s)	0.013	[0.007, 0.019]
Straight men (b/s)	0.008	[0.003, 0.014]
Straight women (g/s)	0.008	[0.002, 0.014]
Straight women (l/s)	0.021	[0.016, 0.027]
Straight women (b/s)	0.012	[0.006, 0.017]
Younger people	−0.009	[−0.015, −0.003]

Outgroup+ refers to positive outgroup evaluations. Ingroup− refers to negative ingroup evaluations. Positive values reflect larger outgroup+ estimates, and negative values reflect larger ingroup− estimates. If the 95% BCI does not include zero, the outgroup+ and ingroup− parameters are reliably different from one another. Thus, constraint tests like the ones reported for the exploratory samples (Tables 2 and 4) are unnecessary. “b/s” refers to an IAT with gay and lesbian stimuli along with straight stimuli; “g/s” refers to an IAT with gay/straight stimuli; “l/s” refers to an IAT with lesbian/straight stimuli.

### Implicit Outgroup Bias.

#### Lower-status groups.

##### Race.

###### Asian vs. White

In both the exploratory and confirmatory samples, Asian participants’ estimates of White-pleasant associations were larger than their estimates of Asian-unpleasant associations. This difference was reliable in the exploratory but not confirmatory sample. In both samples, White-pleasant and Asian-unpleasant parameter estimates were both different from zero. Thus, Asian participants’ outgroup bias reflected stronger positive outgroup evaluations than negative ingroup evaluations, although positive outgroup and negative ingroup evaluations each contributed to outgroup bias.

###### Black vs. White

In both the exploratory and confirmatory samples, Black participants’ estimates of White-pleasant associations were reliably larger than their estimates of Black-unpleasant associations. In both samples, White-pleasant and Black-unpleasant parameter estimates were both different from zero. Thus, Black participants’ outgroup bias reflected stronger positive outgroup evaluations than negative ingroup evaluations, although positive outgroup and negative ingroup evaluations each contributed to outgroup bias.

##### Sexual orientation.

In both the exploratory and confirmatory samples, gay and lesbian participants’ estimates of straight-pleasant associations were larger than estimates of gay/lesbian-unpleasant associations. In both samples, straight-pleasant parameter estimates were different from zero. Gay/lesbian-unpleasant parameter estimates were also different from zero in the exploratory sample, among gay men in the confirmatory sample who completed an IAT with lesbian stimuli, and among lesbian women in the confirmatory sample who completed an IAT with gay stimuli. However, in the confirmatory sample, gay/lesbian-unpleasant parameter estimates were not different from zero among gay men who completed an IAT with gay stimuli, among lesbian women who completed an IAT with lesbian stimuli, and among both gay men and lesbian women who completed an IAT with both gay and lesbian stimuli. Thus, gay and lesbian participants’ outgroup bias reflected stronger positive outgroup evaluations than negative ingroup evaluations, and consistently reflected the contributions of positive outgroup evaluations. Moreover, as the confirmatory analyses reveal, gay and lesbian participants’ outgroup bias did not reflect negative evaluations when their own gender was reflected in the target stimuli in the IAT.

##### Age.

In both the exploratory and confirmatory samples, older participants’ estimates of young-pleasant associations were reliably larger than their estimates of old-unpleasant associations. In both samples, young-pleasant and old-unpleasant parameter estimates were both different from zero. Thus, older participants’ outgroup bias reflected stronger positive outgroup evaluations than negative ingroup evaluations, although positive outgroup and negative ingroup evaluations each contributed to outgroup bias.

#### Higher-status groups.

##### Race.

###### Asian vs. White

In the confirmatory sample, White participants’ estimates of White-unpleasant associations were reliably larger than their Asian-pleasant associations. Their White-unpleasant parameter estimates were reliably different from zero, but their Asian-pleasant associations were not different from zero. Thus, White participants’ outgroup bias reflected stronger negative ingroup evaluations than positive outgroup evaluations, and only negative ingroup evaluations contributed to outgroup bias.

###### Black vs. White

In both the exploratory and confirmatory samples, White participants’ estimates of White-unpleasant associations were reliably larger than their Black-pleasant associations. In both samples, their estimates of White-unpleasant associations were different from zero. In the confirmatory sample, their Black-pleasant associations were different from zero, but were not different from zero in the exploratory sample. Thus, White participants’ outgroup bias reflected stronger negative ingroup evaluations than positive outgroup evaluations, and negative ingroup evaluations consistently contributed to their outgroup bias, but positive outgroup evaluations inconsistently contributed to their bias.

##### Sexual orientation.

In the exploratory sample, straight participants’ estimates of gay/lesbian-pleasant associations were not different from estimates of straight-unpleasant associations, and neither parameter estimate was different from zero. However, in the confirmatory sample, straight participants’ estimates of gay/lesbian-pleasant associations were reliably larger than their straight-unpleasant associations. Their gay/lesbian-pleasant and straight-unpleasant parameter estimates were both different from zero. Thus, straight participants’ outgroup bias reflected stronger positive outgroup evaluations than negative ingroup evaluations, although positive outgroup and negative ingroup evaluations each contributed to outgroup bias, but only in the confirmatory sample, in which we separately modeled the IAT as a function of its stimuli.

##### Age.

In both the exploratory and confirmatory samples, younger participants’ estimates of old-pleasant associations were reliably smaller than their estimates of young-unpleasant associations. In both samples, old-pleasant and young-unpleasant parameter estimates were both different from zero. Thus, younger participants’ outgroup bias reflected stronger negative ingroup evaluations than positive outgroup evaluations, although positive outgroup and negative ingroup evaluations each contributed to outgroup bias.

#### Summary.

Across both exploratory and confirmatory samples, the implicit outgroup biases of lower-status groups (i.e., Asian, Black, gay and lesbian, older) consistently reflected the greater contribution of positive outgroup evaluations than negative ingroup evaluations. However, the implicit outgroup biases of higher-status groups (i.e., White, straight, younger) demonstrated a less consistent pattern of results. The implicit outgroup biases of straight participants reflected the greater contribution of positive outgroup evaluations than negative ingroup evaluations, but the implicit outgroup biases of White and younger participants reflected the greater contribution of negative ingroup evaluations than positive outgroup evaluations. Thus, our findings reveal a positive–negative asymmetry effect for implicit outgroup bias, and primarily in the context of lower-status groups.

### Secondary Analyses.

#### Implicit ingroup bias.

Given the novelty of our process-modeling approach to investigating the contributions of positive and negative evaluations to implicit outgroup bias, we sought to validate this approach by investigating whether it could also reveal the well-established positive–negative asymmetry effect (5)—an ingroup bias that reflects the greater contribution of positive ingroup evaluations than negative outgroup evaluations—that inspired the present research. Using the same methods and criteria as described above, we applied the Quad model to data from participants in the exploratory and confirmatory samples who demonstrated implicit ingroup bias. We summarize the descriptive statistics and *D*-scores of these participants in Table 1.

##### Modeling and model fit.

**Fig. 3.**
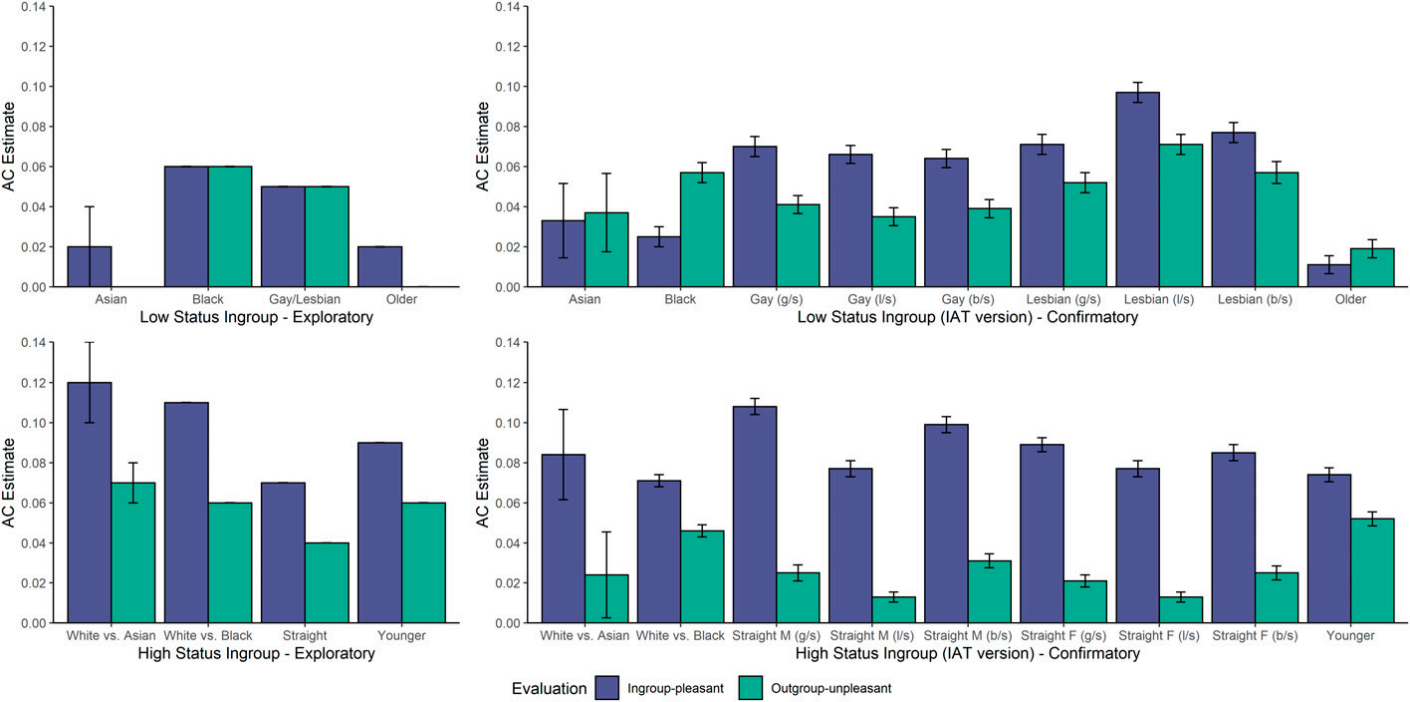
Ingroup-pleasant and outgroup-unpleasant parameter estimates from lower-status participants in the exploratory sample (*Upper Left*), lower-status participants in the confirmatory sample (*Upper Right*), higher-status participants in the exploratory sample (*Lower Left*), and higher-status participants in the confirmatory sample (*Lower Right*). Error bars in the exploratory sample reflect SEs. Error bars in the confirmatory sample reflect 95% BCI.

The specification of the Quad model we applied to the data of participants who demonstrated implicit ingroup bias was largely the same as the specification we applied to the data of participants who demonstrated implicit outgroup bias, with one important difference. Given that the Quad model assumes a compatibility structure to participants’ responses—such that the direction of bias reflects the dominant valences of the underlying associations—we specified one AC parameter to reflect positive evaluations of the ingroup, and another AC parameter to reflect negative evaluations of the outgroup. All other modeling procedures were identical to the outgroup bias analyses. Accounting for sample size, model fit was relatively consistent across datasets, and adequate in all cases (28). We depict parameter estimates in Fig. 3.

##### Planned contrasts.

In order to determine the extent to which positive ingroup versus negative outgroup evaluations contributed to implicit ingroup bias, we conducted a series of planned contrasts to quantify the difference between the ingroup-pleasant AC parameter and the outgroup-unpleasant AC parameter using the same procedures as used with the outgroup bias analyses. We report planned contrasts for the exploratory and confirmatory samples in Tables 4 and 5, respectively.

**Table 4.** Summary of AC parameter comparisons for ingroup-favoring participants in the exploratory sample

Sample	Ingroup+ vs. Outgroup−			Ingroup+ vs. 0			Outgroup− vs. 0		
	Δχ^2^	*P*	*w*	Δχ^2^	*P*	*w*	Δχ^2^	*P*	*w*
Lower-status ingroups									
Asian people	5.35	0.021	0.03	7.10	0.008	0.04	0.00	1.00	0.00
Black people	1.31	0.253	0.00	7,583.65	<0.001	0.07	7,225.38	<0.001	0.07
Gay/lesbian people	101.53	<0.001	0.01	13,894.65	<0.001	0.06	8,176.40	<0.001	0.05
Older people	9.93	0.002	0.03	17.30	<0.001	0.03	0.00	1.00	0.00
Higher-status ingroups									
White (vs. Asian) people	3.84	0.009	0.04	77.37	<0.001	0.14	27.70	<0.001	0.08
White (vs. Black) people	21,225.56	<0.001	0.03	350,610.45	<0.001	0.12	123,738.04	<0.001	0.07
Straight people	28,066.69	<0.001	0.03	311,293.00	<0.001	0.09	116,754.00	<0.001	0.05
Younger people	5,931.24	<0.001	0.03	99,952.27	<0.001	0.11	37,600.03	<0.001	0.07

Ingroup+ refers to positive ingroup evaluations. Outgroup− refers to negative outgroup evaluations.

**Table 5.** Summary of AC parameter comparisons for ingroup-favoring participants in the confirmatory sample

Sample	Ingroup+ vs. Outgroup−	95% BCI
Lower-status ingroups		
Asian people	−0.004	[−0.031, 0.022]
Black people	−0.032	[−0.038, −0.026]
Gay men (g/s)	0.029	[0.023, 0.035]
Gay men (l/s)	0.030	[0.024, 0.036]
Gay men (b/s)	0.025	[0.019, 0.030]
Lesbian women (g/s)	0.019	[0.013, 0.025]
Lesbian women (l/s)	0.026	[0.020, 0.031]
Lesbian women (b/s)	0.021	[0.015, 0.027]
Older people	−0.007	[−0.013, −0.002]
Higher-status ingroups		
White (vs. Asian) people	0.060	[0.029, 0.089]
White (vs. Black) people	0.024	[0.020, 0.029]
Straight men (g/s)	0.083	[0.078, 0.088]
Straight men (l/s)	0.063	[0.059, 0.068]
Straight men (b/s)	0.068	[0.063, 0.073]
Straight women (g/s)	0.068	[0.063, 0.072]
Straight women (l/s)	0.063	[0.059, 0.068]
Straight women (b/s)	0.060	[0.056, 0.065]
Younger people	0.022	[0.018, 0.026]

Ingroup+ refers to positive ingroup evaluations. Outgroup− refers to negative outgroup evaluations. Positive values reflect larger ingroup+ estimates, and negative values reflect larger outgroup− estimates. If the 95% BCI does not include zero, the Ingroup+ and Outgroup− parameters are reliably different from one another. Thus, constraint tests like the ones reported for the exploratory samples (Tables 2 and 4) are unnecessary. “b/s” refers to an IAT with gay and lesbian stimuli along with straight stimuli; “g/s” refers to an IAT with gay/straight stimuli; “l/s” refers to an IAT with lesbian/straight stimuli.

#### Lower-status groups.

##### Race.

###### Asian vs. White

In the exploratory sample, Asian participants’ estimates of Asian-pleasant associations were reliably larger than their estimates of White-unpleasant associations. Their estimates of Asian-pleasant associations were different from zero, but their estimates of White-unpleasant associations were not different from zero. However, in the confirmatory sample, Asian participants’ Asian-pleasant associations were descriptively (but not reliably) smaller than their estimates of White-unpleasant associations. Their estimates of Asian-pleasant associations and White-unpleasant associations were both different from zero. Thus, Asian participants’ ingroup bias reflected positive ingroup evaluations, but inconsistently reflected negative outgroup evaluations.

###### Black vs. White

In the exploratory sample, Black participants’ estimates of Black-pleasant associations were not different from their estimates of White-unpleasant associations. Their estimates of Black-pleasant and White-unpleasant associations were both different from zero. However, in the confirmatory sample, Black participants’ Black-pleasant associations were reliably smaller than their estimates of White-unpleasant associations. Their estimates of Black-pleasant and White-unpleasant associations were both different from zero. Thus, Black participants’ ingroup bias reflected both positive ingroup and negative outgroup evaluations, although the primacy of one type of evaluation was inconsistent across samples.

##### Sexual orientation.

In both exploratory and confirmatory samples, gay and lesbian participants’ estimates of gay/lesbian-pleasant associations were reliably larger than their estimates of straight-unpleasant associations. Their estimates of gay/lesbian-pleasant and straight-unpleasant associations were both different from zero. Thus, gay and lesbian participants’ ingroup bias reflected stronger positive ingroup evaluations than negative outgroup evaluations, although positive ingroup and negative outgroup evaluations each contributed to ingroup bias.

##### Age.

In the exploratory sample, older participants’ estimates of older-pleasant associations were reliably larger than their estimates of younger-unpleasant associations. Their estimates of older-pleasant associations were different from zero, but their estimates of younger-unpleasant associations were not different from zero. However, in the confirmatory sample, older participants’ older-pleasant associations were reliably smaller than their estimates of younger-unpleasant associations. Their estimates of older-pleasant associations and younger-unpleasant associations were both different from zero. Thus, older participants’ ingroup bias reflected positive ingroup evaluations, but inconsistently reflected negative outgroup evaluations.

#### Higher-status groups.

##### Race.

###### Asian vs. White

In both the exploratory and confirmatory samples, White participants' estimates of White-pleasant associations were reliably larger than their estimates of Asian-unpleasant associations. Their estimates of White-pleasant associations and Asian-unpleasant associations were both different from zero. Thus, White participants’ ingroup bias reflected stronger positive ingroup evaluations than negative outgroup evaluations, although positive ingroup and negative outgroup evaluations each contributed to ingroup bias.

###### Black vs. White

In both the exploratory and confirmatory samples, White participants’ estimates of White-pleasant associations were reliably larger than their estimates of Black-unpleasant associations. Their estimates of White-pleasant associations and Black-unpleasant associations were both different from zero. Thus, White participants’ ingroup bias reflected stronger positive ingroup evaluations than negative outgroup evaluations, although positive ingroup and negative outgroup evaluations each contributed to ingroup bias.

##### Sexual orientation.

In both exploratory and confirmatory samples, straight participants’ estimates of straight-pleasant associations were reliably larger than their estimates of gay/lesbian-unpleasant associations. Their estimates of straight-pleasant and gay/lesbian-unpleasant associations were both different from zero. Thus, straight participants’ ingroup bias reflected stronger positive ingroup evaluations than negative outgroup evaluations, although positive ingroup and negative outgroup evaluations each contributed to ingroup bias.

##### Age.

In both exploratory and confirmatory samples, younger participants’ estimates of younger-pleasant associations were reliably larger than their estimates of older-unpleasant associations. Their estimates of younger-pleasant and older-unpleasant associations were both different from zero. Thus, younger participants’ ingroup bias reflected stronger positive ingroup evaluations than negative outgroup evaluations, although positive ingroup and negative outgroup evaluations each contributed to ingroup bias.

##### Summary.

We found evidence for the positive–negative asymmetry effect (5) in all of the higher-status groups in both the exploratory and confirmatory samples, such that their implicit ingroup biases reflected the greater contribution of positive ingroup evaluations than negative outgroup evaluations. However, evidence for the positive–negative asymmetry effect was inconsistent across lower-status groups. The implicit ingroup biases of gay and lesbian participants in both samples consistently reflected the greater contribution of positive ingroup evaluations than negative outgroup evaluations. However, the pattern of evaluations was mixed for Asian, Black, and older participants.

#### Explicit bias.

The positive–negative asymmetry effect (5) was originally identified in the context of ingroup bias as reflected in explicit measures. Although outgroup bias is much less likely to appear on explicit than implicit measures (e.g., refs. 12 and 15), we nevertheless investigated the extent to which explicitly measured intergroup bias reflects the contributions of positive versus negative evaluations.

##### Participants and materials.

Participants in the confirmatory sample each completed two feeling thermometers, one measuring evaluations of their ingroup and another measuring evaluations of the outgroup. Participants responded using an 11-point scale ranging from 0 to 10, such that responses above 5 reflect positive evaluations, responses below 5 reflect negative evaluations, and responses at 5 reflect neutral evaluations. We defined participants as demonstrating outgroup bias when they responded with higher values on the outgroup thermometer than on the ingroup thermometer. We defined participants as demonstrating ingroup bias when they responded with higher values on the ingroup thermometer than on the outgroup thermometer. We did not examine participants who responded with equivalent values on the ingroup and outgroup thermometers. We also did not examine participants in the exploratory samples because we did not measure their explicit biases.

##### Results.

We summarize mean responses on each thermometer (ingroup, outgroup) in Table 6. We also report effect sizes comparing the two thermometers, as well as comparing each thermometer to the scale midpoint. In the majority of samples, both ingroup and outgroup evaluations were reliably above the midpoint of the scale; regardless of their relative bias or group membership, participants on average reported warmth toward both groups across comparisons. The exception to this pattern of results were Asian participants, who reported neutral evaluations of the less-favored group. However, no sample of participants reported reliably negative explicit evaluations of either the ingroup or outgroup. Taken together, these findings dovetail with our findings based on implicit intergroup bias, and with the positive–negative asymmetry effect more generally: regardless of the direction of intergroup bias (ingroup bias, outgroup bias), and regardless of group status (higher, lower), explicit intergroup bias reflects positive evaluations of the favored group rather than negative evaluations of the disfavored group.

**Table 6.** Descriptive statistics by participant sample as a function of group status and direction of explicit bias

Sample	*n* (% of total)	Ingroup warmth		Outgroup warmth		Comparison Cohen’s *d*
		Mean (SD)	Cohen’s *d*	Mean (SD)	Cohen’s *d*	
Lower-status ingroups						
Biased toward outgroup						
Asian people	20 (9.7)	4.75 (1.55)	0.16	7.00 (1.30)	1.54*	1.57*
Black people	796 (15.9)	6.21 (1.78)	0.68*	8.25 (1.39)	2.34*	1.28*
Gay men	830 (5.5)	5.81 (2.11)	0.38*	7.87 (1.75)	1.64*	1.06*
Lesbian women	469 (3.1)	6.64 (1.90)	0.86*	8.52 (1.39)	2.53*	1.13*
Older people	854 (17.1)	6.68 (1.74)	0.97*	8.48 (1.24)	2.81*	1.89*
Biased toward ingroup						
Asian people	144 (69.6)	8.24 (1.34)	2.42*	4.91 (1.79)	0.05	2.11*
Black people	1,658 (33.2)	8.94 (1.28)	3.08*	5.74 (2.03)	0.36*	1.89*
Gay men	10,546 (70.3)	8.82 (1.33)	2.88*	5.45 (2.18)	0.21*	1.87*
Lesbian women	9,923 (66.2)	9.53 (0.85)	5.31*	6.99 (1.70)	1.17*	1.89*
Older people	1,136 (22.7)	8.61 (1.18)	3.05*	6.68 (1.71)	0.98*	1.31*
Higher-status ingroups						
Biased toward outgroup						
White (vs. Asian) people	35 (34.7)	5.43 (2.40)	0.18	8.11 (1.81)	1.72*	1.26*
White (vs. Black) people	702 (14.0)	5.85 (1.83)	0.46*	8.14 (1.45)	2.17*	1.39*
Straight men	1,502 (13.3)	5.65 (1.90)	0.34*	7.56 (1.6)	1.60*	1.09*
Straight women	1,399 (9.3)	6.27 (1.91)	0.67*	8.16 (1.55)	2.04*	1.09*
Younger people	1,597 (31.9)	5.91 (1.66)	0.55*	8.22 (1.30)	2.48*	1.55*
Biased toward ingroup						
White (vs. Asian) people	15 (14.9)	7.73 (1.28)	2.14*	5.73 (1.62)	0.45	1.37*
White (vs. Black) people	991 (19.8)	8.28 (1.31)	2.50*	6.40 (1.68)	0.83*	1.24*
Straight men	5,524 (36.8)	7.92 (1.74)	1.68*	4.58 (2.44)	0.17*	1.57*
Straight women	4,591 (30.6)	8.60 (1.45)	2.48*	5.96 (2.20)	0.44*	1.42*
Younger people	1,221 (24.4)	8.10 (1.38)	2.25	6.00 (1.81)	0.55*	1.30*

For effect size calculations, sample means were compared to scale midpoint (5 on a 0 to 10 scale) in one-sample *t* tests and ingroup and outgroup means were compared to one another in paired samples *t* tests. Gay men were the comparison outgroup for straight men, and lesbian women were the comparison outgroup for straight women (and vice versa).

^*^Effects are reliably different from zero at *P* < 0.01.

## Discussion

The present research examined the extent to which negative ingroup and positive outgroup evaluations contribute to implicit outgroup bias. In contrast to early intergroup relations theories that assumed internalized inferiority and “group self-hatred” among low-status group members (1, 8–10), a consistent pattern of results emerged here in which the outgroup biases of lower-status group members were characterized by more positive outgroup evaluations than negative ingroup evaluations. However, a more varied pattern of results emerged among the outgroup biases of higher-status group members. The outgroup bias of straight participants was also characterized by more positive outgroup evaluations than negative ingroup evaluations, but only when we accounted for participant gender and IAT stimuli. In contrast, White and younger participants’ outgroup bias was characterized by more negative ingroup than positive outgroup evaluations. Taking these data together, the present research dovetails with previous research on the positive–negative asymmetry effect of ingroup bias (5), and suggests a positive–negative asymmetry effect of outgroup bias, especially among members of lower-status groups.

Although the outgroup biases of the lower-status group members examined here reflect the greater influence of positive outgroup than negative ingroup evaluations, the present research also indicates that these biases are not devoid of ingroup negativity. Indeed, across most lower-status groups examined here, both positive outgroup and negative ingroup evaluations significantly influence participants’ responses, although gay and lesbian participants’ outgroup biases did not reflect the contributions of negative ingroup evaluations when their own gender was reflected as stimuli in the IAT. This finding both supports and extends social dominance (11) and system-justifying (12) perspectives, which suggest that the outgroup biases of low-status group members could reflect negative ingroup and positive outgroup evaluations, but remain largely agnostic on the relative contributions of each type of evaluation. The same pattern of results persisted for members of higher-status groups, whose outgroup biases reflected the contributions of both positive outgroup and negative ingroup evaluations.

Although not the focus of our investigation, our analyses of explicit biases further supported the primacy of positive evaluations in intergroup biases. Across samples and comparisons, explicit biases in favor of both ingroups and outgroups reflected positivity toward the favored group rather than negativity toward the comparison group. However, we observed a more nuanced pattern of results in the context of implicit ingroup bias. The implicit ingroup biases of higher status group members consistently reflected more positivity toward the ingroup than negativity toward the outgroup, but the ingroup biases of lower status groups often reflected contributions of both negative and positive evaluations. This asymmetry in evaluations dovetails with previous work that identifies both lower status and minority status as aggravating factors that increase the relative contribution of negativity to ingroup bias (5). Considered along with our implicit outgroup bias findings, these results offer an intriguing conclusion: biases in favor of higher-status group members, whether ingroup or outgroup biases, primarily reflect positive evaluations of the higher-status group, whereas the primacy of positive evaluations is less consistent in the context of favoritism for lower-status groups. In turn, this pattern of results suggests that bias in favor of lower-status groups reflects relatively idiosyncratic causes, whereas bias in favor of higher-status groups may reflect a common cause (e.g., cultural learning).

### Open Questions.

#### Where do positive outgroup evaluations come from?

The present research revealed that outgroup bias often reflects positive outgroup evaluations. How might people form positive evaluations of outgroup members that are more positive than their evaluations of ingroup members? One possibility is through contact. People who develop strong ties to an outgroup community may grow to prefer members of that group to their own. However, contact is asymmetrical, such that members of minority groups (e.g., Black Americans) often have more contact with members of majority groups (e.g., White Americans) than vice versa, and thus contact may be more likely to explain the positive outgroup evaluations of low-status but not high-status group members. Another possibility is that positive outgroup evaluations are formed through cultural learning; positive depictions of outgroup members in movies, television, and other media would account for the evaluations of both low- and high-status group members. Future research should investigate the relationship between media exposure and outgroup evaluations, which in turn may highlight the value of representation in media.

#### Is outgroup bias fundamentally different from ingroup bias?

The present research on the evaluations underlying outgroup bias was inspired by research on the evaluations that comprise ingroup bias (1–5). We tend to recognize ingroup bias as antiegalitarian. After all, bias that primarily reflects positivity and bias that primarily reflects negativity are both prejudice, and prejudice is a precursor to discrimination (29). For example, field audits in banking routinely report that White people are more likely than equally qualified Black people to be approved for mortgages (30, 31). This disparity may not reflect unfair rejection of qualified Black applicants but, rather, lenient acceptance of underqualified White applicants (30). Field audits in employment also support this perspective: hiring disparities (i.e., hiring White people over equally qualified Black people and Hispanic people) are more often linked to helpful acts directed toward White applicants than to hostile acts directed toward minority applicants (32). This body of research is often cited in the context of ingroup bias (e.g., ref. 4) as evidence of the primacy of ingroup favoritism over outgroup derogation. However, and critically, this body of research often fails to report the group membership of the person who engages in the discriminatory act. Consequently, we cannot assume that such discrimination is solely the result of ingroup bias; outgroup bias can result in the same pattern of discrimination. For example, a hiring manager who is a member of a lower-status group but evaluates the higher-status group positively might engage in preferential treatment of higher-status outgroup applicants over ingroup applicants. The modeling methods used in the present research provides a template—and our findings lay the groundwork—for examining whether outgroup bias in general, and positive outgroup versus negative ingroup evaluations specifically, predict judgments and behavior.

#### Does the composition of higher-status group members' outgroup biases depend on the group?

The most varied outcome that emerged in the present research was among the outgroup biases of higher-status group members. When we separated our analyses by participant gender and IAT stimuli, the outgroup bias of straight participants demonstrated the positive–negative asymmetry effect. However, the outgroup biases of White and younger participants were characterized by more negative ingroup than positive outgroup evaluations. Further research is needed to determine whether this pattern of sexuality evaluations represent the exception or the rule for implicit outgroup biases among higher status group members. Perhaps sexual orientation creates an ingroup that is especially devoid of negative associations: the desire for sexual or romantic relationships that defines group membership is definitively positive. Even straight people who have positive evaluations of gay or lesbian people that outweigh their positive evaluations of other straight people likely have more positive than negative associations with their ingroup, otherwise they would not identify as straight. Other members of higher-status groups with more permeable boundaries than those defined by race and age (e.g., religious or political affiliation) may likewise produce outgroup biases that demonstrate the positive–negative asymmetry effect. Given the paucity of research in this area, the outgroup biases of high-status group members represent a potentially fruitful topic for future investigations.

### Limitations.

The present research is limited in that it only examined outgroup biases in the context of three social identities: race, sexual orientation, and age. Although these identities are highly salient in most intergroup interactions, future research should examine whether the patterns of outgroup bias demonstrated here generalize to other social identities and situations. Outgroup evaluations may be expected to be less positive in contexts with protracted histories of intergroup hostility, such as South Africa, Northern Ireland, and the American South (4), although outgroup bias should be less common in these contexts overall. Similarly, an open question remains as to whether our observed patterns of results will persist outside of predominantly Western, educated, industrialized, rich, and democratic contexts (33). The present research is also limited because it relies only on the IAT as an implicit measure, although this criticism also applies to most of the existing literature on implicit outgroup favoritism. The IAT is a categorization task, and thus necessarily makes social categories salient. Given that implicit bias is moderated by category salience (34), open questions remain as to whether the pattern of results observed here would persist on an implicit measure that does not rely on category labels (e.g., ref. 35), relies on only one target category (e.g., ref. 36), or assesses personalized evaluations (e.g., ref. 37). That said, the present research highlights the utility of using the IAT to study outgroup biases, especially in the context of lower-status groups. The vast majority of lower-status group members demonstrate ingroup rather than outgroup bias on explicit measures (Table 6), but demonstrate a more moderate tendency toward ingroup versus outgroup bias on the IAT (Table 1). Thus, the IAT appears to offer unique insight into the outgroup biases of lower-status group members. The present research is also correlational, so we cannot rule out unobserved third variables that might provide alternate explanations to our observed results. Our highly powered correlational findings would be bolstered by future experimental research that manipulates group status to reveal deeper insight into the processes underlying implicit outgroup bias.

## Conclusions

The present research demonstrates the primacy of positive outgroup over negative ingroup evaluations in the outgroup biases of lower-status group members. In contrast, the outgroup biases of higher-status group members reflect a more varied pattern of positive and negative evaluations. We hope that these findings can be used as a basis for future research that examines the downstream consequences of outgroup bias. Separately studying the positive and negative components of outgroup bias may provide a fruitful means of advancing our understanding of intergroup relations.

## Supplementary Material

Supplementary File

## Data Availability

Data have been deposited in the Open Science Framework (https://osf.io/pf8cd/?view_only=72f94988d0a1497f992a03a71dfb213c). Data and analysis scripts can be found at https://osf.io/pf8cd/?view_only=72f94988d0a1497f992a03a71dfb213c. Materials for the IATs are available at https://osf.io/pf8cd/?view_only=72f94988d0a1497f992a03a71dfb213c (Asian/White) (38); https://osf.io/52qxl/ (Black/White) (39); https://osf.io/ctqxo/ (sexual orientation) (40); and https://osf.io/cv7iq/ (age) (41).

## Footnotes

^*^In this report, we use the term “implicit” to mean “indirect.” Thus, an “implicit measure” assesses mental contents indirectly. We use the term “association” to refer to one of the mental constructs assessed by implicit measures. However, we make no strong assumptions about the representational nature of the constructs assessed by implicit measures.

^†^We deviated from our preregistered *n* = 10,000 participants per sample, analyzing instead *n* = 5,000 participants per sample. Our analytic approach in the confirmatory studies proved to be too computationally intensive to feasibly analyze larger samples.

^‡^Along with two AC parameters, we estimated D, OB, and G parameters for each sample. We do not report these parameters in the main text, as they are not relevant to the present research, but report them in full in *SI Appendix*.

^§^The feedback cut-offs were chosen according to effect size estimates (18); a *D*-score smaller in magnitude than ±0.15 suggests a small effect. We report in *SI Appendix* a series of robustness analyses using different (i.e., more stringent, more lenient) cutoff points. The pattern of results is identical to what we report in the main text.
