# Supplementary material for: The contributions of positive outgroup and negative ingroup evaluation to implicit bias favoring outgroups
Source: Proc Natl Acad Sci U S A. 2022 Sep 26;119(40):e2116924119. doi: 10.1073/pnas.2116924119 (PMC9546602; doi:10.1073/pnas.2116924119)
Supplement: Supplementary File [file pnas.2116924119.sapp.pdf]

The Contributions of Positive Outgroup and Negative Ingroup Evaluation to Implicit Bias Favoring Outgroups

Parameter Estimation

Two association (AC) parameters were estimated for each participant: one AC parameter reflected negative evaluations of the ingroup, and another AC parameter reflected positive evaluations of the outgroup. One each Detection, Overcoming Bias, and Guessing parameter was estimated for each participant. The Guessing parameter was specified such that values >.5 reflected a tendency to respond with the *pleasant* key, values <.5 reflected a tendency to respond with the *unpleasant* key, and values =.5 reflect no response bias. We report only AC parameters in the main text, as they are most relevant to the present research. Here in the Supplementary Materials, we report the remaining Quad model parameters for the exploratory and confirmatory analyses for participants who demonstrate outgroup bias (Table S1) and participants who demonstrate ingroup bias (Table S2).

Table S1.

	Outgroup Bias			*Ingroup Bias*		
Group	*D*	*OB*	*G*	*D*	*OB*	*G*
White (vs. Asian)	NA	NA	NA	.90 (.01)	.67 (.12)	.52 (.04)
Asian	.88 (.01)	.26 (.27)	.56 (.03)	.89 (.01)	.26 (1.13)	.55 (.04)
White (vs. Black)	.86 (.00)	1.00 (.00)	.51 (.00)	.90 (.00)	.87 (.00)	.57 (.00)
Black	.87 (.00)	.79 (.01)	.56 (.00)	.88 (.00)	.79 (.00)	.55 (.00)
Younger	.88 (.00)	1.00 (.08)	.52 (.00)	.91 (.00)	.80 (.00)	.56 (.00)
Older	.90 (.00)	.00 (.55)	.59 (.02)	.95 (.00)	.35 (.00)	.59 (.00)
Heterosexual	.78 (.00)	0*	.55 (.00)	.83 (.00)	.00 (.00)	.52 (.00)
Homosexual	.83 (.00)	.00 (.02)	.53 (.00)	.84 (.00)	.64 (.00)	.58 (.00)

Note: Means and standard errors for Quad parameter estimates for participants in the exploratory sample. *D* = detection of correct response. *OB* = overcoming biased associations when the conflict with correct response. *G* = bias in absence of other guides to response.

*Because of floor effects, standard errors could not be modeled for OB parameters in the case of heterosexual participants demonstrating outgroup bias. Instead, parameters were estimated with OB fixed at .0001.

Table S2.

	Outgroup Bias			Ingroup Bias		
Group	*D*	*OB*	*G*	*D*	*OB*	*G*
Lower Status						
Asian	.91 [.89, .93]	.07 [.00, .32]	.47 [.40, .55]	.91 [.89, .93]	.57 [.06, .97]	.42 [.37, .47]
Black	.95 [.95, .96]	.00 [.00, .01]	.54 [.52, .56]	.94 [.93, .94]	.99 [.96, 1.00]	.55 [.53, .57]
Gay M (g/s)	.92 [.91, .92]	.00 [.00, .02]	.59 [.57, .60]	.92 [.91, .92]	.93 [.75, 1.00]	.63 [.62, .65]
Gay M (l/s)	.92 [.91, .92]	.00 [.00, .01]	.56 [.54, .58]	.91 [.91, .92]	.54 [.14, .90]	.62 [.61, .63]
Gay M (b/s)	.91 [.91, .92]	.00 [.00, .01]	.56 [.55, .58]	.92 [.91, .92]	.60 [.13, .95]	.64 [.63, .65]
Lesbian W (g/s)	.92 [.91, .92]	.00 [.00, .01]	.56 [.54, .57]	.90 [.90, .91]	.97 [.89, 1.00]	.59 [.58, .60]
Lesbian W (l/s)	.91 [.91, .92]	.00 [.00, .02]	.55 [.54, .57]	.91 [.91, .92]	1.00 [1.00, 1.00]	.63 [.62, .64]
Lesbian W (b/s)	.91 [.91, .92]	.00 [.00, .01]	.55 [.54, .57]	.91 [.90, .91]	.99 [.94, 1.00]	.60 [.59, .61]
Younger	.93 [.92, .93]	.77 [.25, .99]	.54 [.52, .56]	.95 [.94, .95]	.01 [.00, .01]	.54 [.52, .55]
Higher Status						
White (vs. Asian)	.97 [.94, .99]	.38 [.01, .92]	.51 [.38, .66]	.93 [.91, .95]	.49 [.06, .93]	.49 [.40, .59]
White (vs. Black)	.94 [.93, .94]	.96 [.86, 1.00]	.51 [.49, .54]	.96 [.96, .97]	.01 [.00, .05]	.56 [.54, .58]
Straight M (g/s)	.90 [.89, .90]	.02 [.00, .06]	.59 [.57, .61]	.92 [.91, .92]	.00 [.00, .03]	.60 [.59, .61]
Straight M (l/s)	.90 [.89, .91]	.02 [.00, .09]	.57 [.56, .59]	.92 [.92, .93]	.00 [.00, .01]	.58 [.56, .59]
Straight M (b/s)	.89 [.89, .90]	.02 [.00, .07]	.59 [.57, .61]	.92 [.92, .92]	.00 [.00, .01]	.57 [.56, .58]
Straight W (g/s)	.90 [.90, .91]	.00 [.00, .03]	.55 [.53, .57]	.93 [.92, .93]	.00 [.00, .00]	.55 [.54, .56]
Straight W (l/s)	.91 [.90, .91]	.00 [.00, .02]	.57 [.55, .58]	.92 [.92, .93]	.00 [.00, .00]	.55 [.54, .57]
Straight W (b/s)	.90 [.90, .91]	.00 [.00, .02]	.55 [.53, .57]	.92 [.92, .93]	.00 [.00, .00]	.53 [.51, .54]
Older	.98 [.97, .99]	.00 [.00, .01]	.62 [.58, .65]	.96 [.96, .96]	.97 [.87, 1.00]	.61 [.58, .65]

Note: Means and 95% Bayesian Confidence Interval for Quad parameter estimates for participants in the confirmatory sample. *D* = detection of correct response. *OB* = overcoming biased associations when the conflict with correct response. *G* = bias in absence of other guides to response. "g/s" refers to an IAT with gay/straight stimuli. "l/s" refers to an IAT with lesbian/straight stimuli. "b/s" refers to an IAT with gay and lesbian stimuli along with straight stimuli.

Table S3.

	Error Rate	χ^2	*p*	*w*
Lower Status Ingroups				
Asian People	7.99%	1.07	.785	0.02
Black People	7.61%	1,862.48	< .001	0.04
Gay/Lesbian People	9.51%	2,740.66	< .001	0.04
Older People	4.71%	20.79	< .001	0.01
Higher Status Ingroups				
White (vs. Black)	7.17%	10,472.09	< .001	0.06
Straight People	11.13%	16,839.71	< .001	0.05
Younger People	6.15%	1,514.96	< .001	0.05

Note: Model fit statistics for the exploratory sample.

Table S4.

Confirmatory Sample	Error Rate	T_1	*p*	*w*	T_2	*p*	*T1 ind.*	*p*
Lower Status Ingroups								
Asian People	6.88%	.26	.030	.005	.43	.212	.09	.468
Black People	5.31%	1.38	< .001	.002	23.86	< .001	.01	.476
Gay Men (g/s)	6.93%	1.95	< .001	.003	19.42	< .001	.03	.471
Gay Men (l/s)	7.14%	5.92	< .001	.005	14.50	< .001	.09	.453
Gay Men (b/s)	7.06%	1.73	< .001	.003	9.89	< .001	.03	.468
Lesbian Women (g/s)	7.54%	1.15	< .001	.002	25.90	< .001	.08	.456
Lesbian Women (l/s)	7.40%	5.66	< .001	.006	19.80	< .001	.04	.468
Lesbian Women (b/s)	7.49%	4.62	< .001	.005	7.59	< .001	.03	.469
Older People	3.57%	.52	< .001	.001	38.36	< .001	.02	.470
Higher Status Ingroups								
White (vs. Asian)	5.28%	.02	.316	.002	1.60	.226	.008	.456
White (vs. Black)	4.82%	.37	< .001	.002	11.60	< .001	.02	.478
Straight Men (g/s)	7.60%	12.30	< .001	.010	24.36	< .001	.12	.445
Straight Men (l/s)	7.60%	12.99	< .001	.010	55.81	< .001	.13	.444
Straight Men (b/s)	7.45%	7.47	< .001	.008	20.97	< .001	.10	.454
Straight Women (g/s)	7.06%	13.89	< .001	.009	32.61	< .001	.06	.457
Straight Women (l/s)	6.95%	13.88	< .001	.010	38.81	< .001	.08	.455
Straight Women (b/s)	6.91%	21.07	< .001	.011	57.87	< .001	.07	.458
Younger People	5.65%	.05	.001	.001	4.26	< .001	.013	.480

Note: Model fit statistics for the confirmatory sample. “g/s” refers to an IAT with gay/straight stimuli. “l/s” refers to an IAT with lesbian/straight stimuli. “b/s” refers to an IAT with gay and lesbian stimuli along with straight stimuli.

Robustness Analyses

In the main text, we report analyses on the exploratory sample based on an outgroup bias threshold based on an IAT *D*-score > |.15|. Recognizing the relatively arbitrary nature of this cutoff, we report in Table S5 a parallel analysis based on a more stringent cutoff (*D* > |.20|), as well as in Table S6 a parallel analysis based on a more lenient cutoff (*D* > |.10|). In both analyses, the pattern of results is the same as reported in the main text.

Table S5.

			Outgroup + vs. Ingroup –			Outgroup + vs. 0			Ingroup – vs. 0		
	N	IAT *D*-score	$\Delta\chi^2$	*p*	*w*	$\Delta\chi^2$	*p*	*w*	$\Delta\chi^2$	*p*	*w*
Lower Status											
Asian	32	.51	3.96	.047	.03	31.86	<.001	.09	9.09	.002	.05
Black	10,932	.48	225.26	< .001	.01	4,707.55	< .001	.06	2,145.65	< .001	.04
Gay/lesbian	14,906	.47	2,982.95	< .001	.04	6,874.30	< .001	.06	160.05	< .001	.01
Older	1,246	.66	51.60	< .001	.02	1,497.90	< .001	.10	884.62	< .001	.08
Higher Status											
White (vs. Black)	20,671	-.42	813.93	< .001	.02	1,107.19	<.001	.02	0.00	.999	.00
Straight	48,888	-.46	0.00	1.00	.00	0.00	1.00	.00	0.00	1.00	.00
Younger	4,019	-.38	76.84	< .001	.01	237.32	< .001	.02	12.56	< .001	.01

Note. Summary of planned contrasts for participants in exploratory sample between Outgroup Positive (+) versus Ingroup Negative (–) AC parameters, and between each AC parameter and zero. In these analyses, outgroup bias was defined as an IAT *D*-score > |.20|.

Table S6.

			Outgroup + vs. Ingroup –			Outgroup + vs. 0			Ingroup – vs. 0		
	N	IAT *D*-score	$\Delta\chi^2$	*p*	*w*	$\Delta\chi^2$	*p*	*w*	$\Delta\chi^2$	*p*	*w*
Lower Status											
Asian	40	.43	3.11	.08	.03	31.21	<.001	.08	10.74	.001	.04
Black	13,734	.41	263.54	< .001	.01	4,800.42	< .001	.05	1,926.60	< .001	.03
Gay/lesbian	19,188	.40	3,739.58	< .001	0.04	7402.50	< .001	0.06	44.82	< .001	.00
Older	1,361	.61	53.64	< .001	0.02	1,460.94	< .001	.09	834.23	< .001	.07
Higher Status											
White (vs. Black)	30,538	-.33	1,087.94	< .001	0.02	1,270.61	<.001	.02	0.00	1.00	.00
Straight	67,540	-.37	0.00	1.00	0.00	0.00	1.00	0.00	0.00	1.00	0.00
Younger	6,048	-.31	104.81	< .001	0.01	277.49	< .001	.02	5.97	.015	.00

Note. Summary of planned contrasts for participants in exploratory sample between Outgroup Positive (+) versus Ingroup Negative (–) AC parameters, and between each AC parameter and zero. In these analyses, outgroup bias was defined as an IAT *D*-score > |.10|.
